# Dynamic Modeling of Cell Migration and Spreading Behaviors on Fibronectin Coated Planar Substrates and Micropatterned Geometries

**DOI:** 10.1371/journal.pcbi.1002926

**Published:** 2013-02-28

**Authors:** Min-Cheol Kim, Devin M. Neal, Roger D. Kamm, H. Harry Asada

**Affiliations:** 1 BioSystem & Micromechanics IRG, Singapore MIT Alliance Research Technology, Singapore; 2 Departments of Mechanical Engineering, Massachusetts Institute of Technology, Cambridge, Massachusetts, United States of America; 3 Biological Engineering, Massachusetts Institute of Technology, Cambridge, Massachusetts, United States of America; University of Notre Dame, United States of America

## Abstract

An integrative cell migration model incorporating focal adhesion (FA) dynamics, cytoskeleton and nucleus remodeling, actin motor activity, and lamellipodia protrusion is developed for predicting cell spreading and migration behaviors. This work is motivated by two experimental works: (1) cell migration on 2-D substrates under various fibronectin concentrations and (2) cell spreading on 2-D micropatterned geometries. These works suggest (1) cell migration speed takes a maximum at a particular ligand density (∼1140 molecules/µm^2^) and (2) that strong traction forces at the corners of the patterns may exist due to combined effects exerted by actin stress fibers (SFs). The integrative model of this paper successfully reproduced these experimental results and indicates the mechanism of cell migration and spreading. In this paper, the mechanical structure of the cell is modeled as having two elastic membranes: an outer cell membrane and an inner nuclear membrane. The two elastic membranes are connected by SFs, which are extended from focal adhesions on the cortical surface to the nuclear membrane. In addition, the model also includes ventral SFs bridging two focal adhesions on the cell surface. The cell deforms and gains traction as transmembrane integrins distributed over the outer cell membrane bond to ligands on the ECM surface, activate SFs, and form focal adhesions. The relationship between the cell migration speed and fibronectin concentration agrees with existing experimental data for Chinese hamster ovary (CHO) cell migrations on fibronectin coated surfaces. In addition, the integrated model is validated by showing persistent high stress concentrations at sharp geometrically patterned edges. This model will be used as a predictive model to assist in design and data processing of upcoming microfluidic cell migration assays.

## Author Summary

Cell migration is a complex, multifaceted process, triggered by chemotaxis and haptotatic responses from the extracellular matrix (ECM). It is triggered by a thin lamellipodium protrusion at the leading edge, followed by the assembly of a number of focal adhesions between the lamellipodium base and the ECM. Afterwards, actin stress fibers extend from nascent focal adhesions, some of which connect to the nucleus. In this work, we have developed a dynamic model of cell migration incorporating these four mechanisms of cell biology, such as remodeling of cell and nuclear membranes, focal adhesion dynamics, actin motor activity, and lamellipodia protrusion at the leading edge. We successfully compared our model with existing experimental works of cell migration on (1) substrates with various fibronectin coating concentrations, and (2) cell spreading on three patterned surfaces. Finally, our model demonstrates how actin stress fibers anchored at the trailing edge play a key role, leading to an increase in cell migration speed. Thereby, the model will not only provide new insights on better building such an experiment, but also further experiments will allow us to better validate the model.

## Introduction

Understanding cell migration mechanisms is a critical issue in many biophysical phenomena, including angiogenesis, tumor growth, metastasis, and wound healing [1]–[3]. Cell migration is a complex multifaceted process, triggered by chemotaxis and haptotatic responses from the extracellular environment [4]. Initially, a thin lamellipodium protrudes due to actin polymerization at the leading edge, followed by actin depolymerization at the lamellipodium base [5]–[8]. Focal adhesions (FAs) are assembled between the lamellipodium base and the extracellular matrix (ECM). FAs are composed of FA molecules (such as FAK, paxillin, vinculin, Zyxin, VASP, and talin), and transmembrane proteins, especially integrins α_v_β_3_ and α_v_β_5_ that link the ECM to the cytoskeleton via FA molecules [9], [10]. Afterwards, contractile bundles of actin filaments, called stress fibers (SFs), extend from nascent FAs and some of which connect to the nucleus [11]. The corresponding motor activity exerts force on the FA's fore and aft [12], enabling the generation of a traction force and the release of FAs in the rear of the cell, creating the cell body's forward movement.

The following individual processes of these steps of cell migration have been studied extensively in the literature: actin polymerization and depolymerization [6]–[8], focal adhesion dynamics [13], [14], and motor activity of contractile myosin [15], [16]. Furthermore, both experiments and computational models from those prior works mostly involve 2-dimensional migration on a flat substrate. However, it still remains a challenge to elucidate how these mechanisms work together to mimic 2-D cell migratory behaviors, which have been observed in existing experimental works. The current work is motivated by two experimental works; one on Chinese Hamster Ovary (CHO) cell migration on 2-D (Figure S1-A) fibronectin coated substrate [17], and the other on cells spreading on 2-D (Figure S1-B) fibronectin coated micropatterns on chips [18]. Cell migration experiments have indicated that three separate variables, such as substratum ligand density, cell integrin expression level and integrin–ligand binding affinity, significantly affect changes in cell migration speed. For example, when cells migrate on various fibronectin coating concentrations, the cell migration speed takes a maximum at a particular ligand density (∼1140 molecules/µm^2^) with a biphasic curve [17]. On the other hand, cell spreading experiments have revealed that interactions between a cell's cytoskeleton and micropatterned geometries impinge on cell morphology and mechanics [18]. For example, when cell spreading occurs on a crossbow pattern, the cell exhibits locally high traction forces at three corners of the pattern, which may be due to concentrated ventral SFs.

Explaining complex interactions with 3-D ECM structure (Figure S1-C&D) entails a proper model mechanism of cell spreading because the cell morphology in 3-D ECM is strikingly different from that on 2-D ECM surfaces as the cell is elongated with the highest directionality and highest velocity of migration in 3-D ECM, but the cell forms peripheral lamellae with an increased random migration on 2-D plastic or fibronectin-coated substrates [19]. To this end, we have built a computational 3-D cell migration model on 2-D curved ECM surfaces and discovered that the cell migration speed differs depending on the diameter of a sprout, and explained the mechanism [20]. It is interesting to note that there is an optimal sprout diameter that creates the highest speed of cell migration. In a similar way as on 2-D curved surfaces, we first aim to look at 3-D cell migration model on 2-D planar surfaces with various fibronectin coating concentrations to understand relationship between the migratory speed and ligand surface density. After verifying our 3-D model with 2-D cell migratory mechanism, we then aim to look at 3-D cell spreading model on various 2-D fibronectin-coated patterns. This entails a) deformation mechanics of both cell membrane and nucleus, b) 3-D interactions between transmembrane integrins and ECM ligands, leading to focal adhesion formation, c) SF formation and traction generation, and d) lamellipodium protrusion at the leading edge of the cell. Integration of these key mechanisms is pivotal for elucidating the aforementioned migratory and spreading behaviors.

Several prior works have incorporated multiple force-generating systems in their cell migratory models 21–23. These works, however, have considered only frictional forces with the substrate rather than focal adhesion (FA) dynamics [24], [25], which generate a mechanical traction force due to a gradient in degraded ligand matrix density during the formation and rupture of ligand-receptor bonds [13], [24], interplay between Rac-mediated membrane protrusion and adhesions at the leading edge [25]. To explain these mechanisms, a model having ligand-receptor bonds distributed across the cell membrane is necessary. Thereby, we have applied FA dynamics to our cell migratory model. Furthermore, our 3-D computational cell spreading model differs from other existing 2-D models [26]–[29] in that we incorporate aforementioned FA dynamics, cell membrane and nuclear remodeling, actin motor activity, and lamellipodia protrusion. Additionally, our model can predict 3-D spatiotemporal behavior of cell spreading on 2-D micropatterns as well as spatiotemporal distribution of two kinds of actin stress fibers (SFs), one is a SF connected to the nucleus and the other is a ventral SF, in 3-D intracellular domain.

To our knowledge, neither a cell migration or a spreading model integrating focal adhesion dynamics, cell membrane and nuclear remodeling, actin motor activity, and lamellipodia protrusion has been published that reflects 3-D spatiotemporal dynamics of both cell spreading and migration, all interfaced with a 2-D planar surface and fibronectin coated patterns. In the following, numerical simulations demonstrate the diverse migration and spreading behaviors in relation to the various ligand densities of migrating 2-D surfaces and micropatterns, respectively.

## Results

First, we aim to verify our model against 2-D cell migration on fibronectin coated substrates under five different fibronectin coating concentrations [18]. After this verification, we further aim to verify our model against 2-D cell spreading on micro-patterned structures. We simulate binding kinetics between integrin receptors and extracellular matrix protein ligands (eg. collagen, fibronectin and laminin), model the formation of SFs, and predict how the forces acting on the cell deform the nucleus and the cytoskeleton, resulting in diverse patterns of the cell profile and migratory motion. Simulations of cell migration and spreading were performed respectively for five different ligand surface densities on the planar surface and three different fibronectin coated micropatterns. Fibronectin was considered for both those two sets of simulations. Fibronectin ligand surface densities are summarized in Table 1.

**Table 1:** Ligand surface density (Fibronectin).

	Cell migration					Cell spreading
Plating concentration [µg/mL]^a^	1	10	30	60	80	25
Ligand surface density [molecules/µm^2^]	19.4	192	568	1140	1522	475

a The molecular mass of Fibronectin is 480 kDa, the corresponding ligand surface density was converted using the relationship between plating concentration and ligand surface density of Fibronectin [41].

At the initial state of each simulation, both cell and nuclear membranes were assumed to be round. Since the migration model is stochastic, simulations were repeated multiple times from the same initial conditions. Table 2 lists all the parameters used for the simulations with numerical values and their sources.

**Table 2:** List of simulation parameters.

Parameter	Definition	Value	Sources
*A*	Area [µm^2^]		
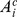	Area of the *i*-th surface of the cell membrane [µm^2^]		
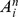	Area of the *i*-th surface of the nucleus [µm^2^]		
*A_L_*	Equilateral triangular area of ligands surface element [µm^2^]	0.243	Current work
*A_SF_*	Averaged SFs' sectional area [µm^2^]	0.196	[60]
*C_c_*	Friction coefficients associated with the energy dissipation at the integrin node [N s m^−1^]	0.001	[21], [32]
*C_n_*	Friction coefficients associated with the energy dissipation at the nuclear node [N s m^−1^]	0.001	[21], [32]
*F*	Force [N]		
*E*	Elastic energy [pJ]		
*E_SF_*	Young's modulus value of SFs [kPa]	230	[59]
*L*	Length		
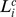	Length of the *i*-th line on the surface of the cell membrane [µm]		
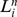	Length of the *i*-th line on the surface of the nucleus [µm]		
*L_b_*	Stretched length of bonds between receptors and ligands		
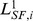	Length of the *i*-th single unit of SFs at the present time [nm]		
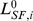	Length of the *i*-th single unit of SFs at the previous time [nm]		
*_N_*	Number of nodes at each membrane	549	Current work
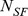	Number of contractile compartments in the *i*-th SFs		
*P*	Probability		
*W*	Total stored elastic energy		
*c_L_*	Ligand density on the lumen [molecule µm^−2^]		
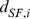	Distance between *i*-th integrin and *j*-th nuclear nodes		
*h_c_*	Critical height [nm]	300	Current work
*h_p_*	Height from the surface to the *i*-th integrin node [nm]		
*k_f_*	Forward reaction rate [molecule^−1^ s^−1^]	1.0	Current work
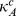	Effective spring constant of area elements of the cell membrane [N/m]	1.0×10^−4^	[52]
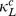	Effective spring constant of line elements of the cell membrane [N/m]	5.0×10^−5^	[32], [54]
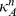	Effective spring constant of area elements of the nucleus [N/m]	1.0×10^−4^	[52]
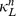	Effective spring constant of line elements of the nucleus [N/m]	5.0×10^−3^	[53]
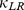	Effective spring constant of ligand-receptor bond [pN/nm]	1.0	[40]
*k_on_*	Kinetic association rate [s^−1^]		
*k_off_*	Kinetic dissociation rate [s^−1^]		
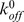	Kinetic dissociation rate at an unstressed state [s^−1^]		Current work
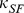	Effective stiffness of the *i*-th single unit of SFs [N/m]		
*n_b_*	Number of bonds between receptors and ligands		
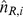	Unit normal vector at the *i*-th integrin node		
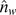	Unit normal vector at the local surface of the lumen		
*t*	Time [s]		
***v***	Velocity vector [nm/s]		
*v_m_*	Sliding rate of non-muscle myosin II on the actin filaments [nm/s]		[61]–[63]
***x***	Location vector [µm]		
*x_L,i_*	Root of ligand-receptor bonds on the local surface of the lumen [nm]		
***λ***	Equilibrium distance of an integrin [nm]	30	[38]
**Sup**			
*D*	Drag or friction		
*E*	Elastic		
*FA*	Focal adhesion		
*SF*	Stress fiber		
*c*	cytoskeleton		
*n*	nucleus		
*i*	*i*-th node		
*0*	Previous time or initial state		
*1*	Present time		
**Sub**			
*b*	bond		
*r*	rupture		

### Integrated cell migration model

**Figure 1:**
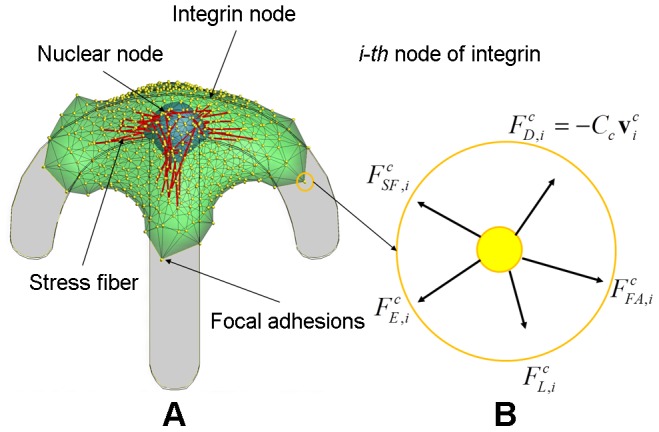
Dynamic model of cell migration. A) Integrated cell migration model consisting of the cytoskeleton, the nucleus, *N* integrin nodes on the surface of cytoskeleton, *N* nuclear nodes on the surface of nucleus, and two types of actin SFs which connect the integrin node to the nuclear node and between integrin nodes; a top view of the model showing triangular mesh network of double membranes of cytoskeleton and nucleus. B) the free body diagram of the i-th integrin node in the circle marked in A) where five external forces are acting. Note that, while shown in 2-D, the force balance exists in 3-D.

**Figure 2:**
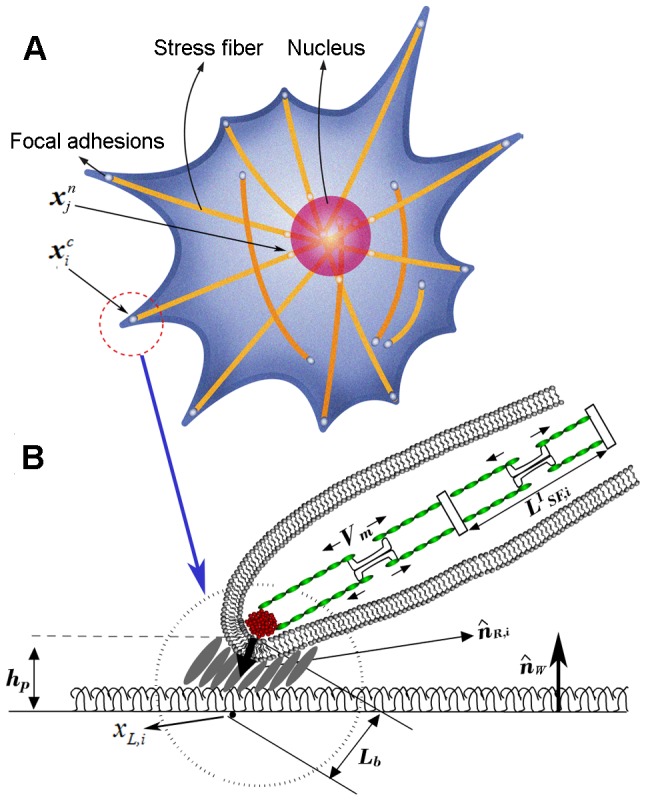
Incorporation of key mechanisms of cell biology. 3-D integrated cell migration model A) schematic representation of cell migration model on the planar substrate, showing deformable cell and nuclear membranes, focal adhesions, and actin SFs, B) a magnified view in A) showing the structure of focal adhesion including the attachment of the end of SFs through an integrin node to the underlying extracellular matrix, illustrating a stochastic ligand-receptor bonding process at the focal adhesion site, and showing the structure of actin SFs. Note that, A) and B) represent top and side views, respectively.

We model the geometric structure of a cell as a double mesh structure: the outer mesh representing the cell membrane and the inner mesh for the nucleus membrane. See Figure 1-A. Each mesh consists of *N* nodes connected elastically to adjacent nodes, forming a double elastic membrane. The inner and outer mesh nodes may be connected when SFs are formed between the nucleus and the cell membrane [30], [31]. Multiple transmembrane integrins are bundled together and placed at each node on the outer mesh. They can bind to ligands on the matrix substrate, forming a focal adhesion, to which a SF is connected (Figure 2-A). Furthermore, the model also includes ventral SFs which extend between two focal adhesions.

Figure 1-B shows the free body diagram of the *i*-th node of the cytoskeleton, called the *i*-th integrin node, where a bundle of integrins is formed. Double membranes in the integrated cell migration model move in Lagrangian approach. Acting on this node are force vectors due to frictional dissipative force 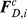, focal adhesion force 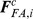, elastic energy force 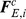, SF force 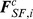, and lamellipodium force 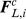. The equation of motion for each integrin node is given by
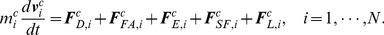
(1)where 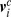 is the velocity vector of the *i*-th integrin node. Similarly, the equation of motion for each node of the nucleus is given by
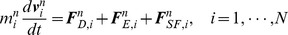
(2)where 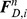, 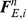 and 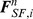 are frictional dissipative force, elastic energy force and SF force at the *i*-th nuclear node, respectively, and 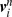 is the velocity of the *i*-th nuclear node. The velocities 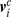 and 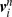 are expressed as
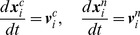
(3)where 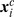 and 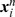 represent coordinates of the *i*-th integrin node and the *i*-th nuclear node, respectively.

Most of the frictional dissipative term 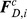 arises from the rupture of stretched ligand-receptor bonds; when they rupture, the stored strain energy is released and dissipated. Similarly, 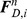 also arises from the energy stored in SFs that, when F-actin is depolymerized, the stored strain energy is released and dissipated. These dissipative forces can be written as
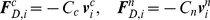
(4)where 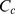 and 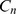 are friction coefficients associated with the energy dissipation at the integrin node and the nuclear node, respectively. In the literature these coefficients are estimated as 0.001 Ns/m [21], [32], [33]. 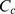 comes from the binding and rupture of ligand-receptor bonds and cannot easily be measured [34].

It should be noted that the sum of forces is zero because the motion is quasi-static in time (Text S1, Figure S2), thus Equations (1)–(4) can be simplified to the following two force balance equations:
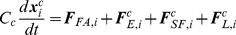
(5)

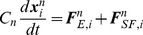
(6)


### Focal adhesion dynamics

Formation of a focal adhesion is described by a stochastic process due to binding kinetics between receptors and ligands on the surface of ECM. Monte Carlo simulation methods have been established for various ligand-receptor binding kinetics in the literature [35]–[37]. We apply a similar technique to cell migration and spreading on planar surfaces. First we represent the 2-D planar surface and a micropatterned geometry using a mesh of triangles, over which ligands are distributed (Figure S3). Each focal adhesion consists of a bundle of ligand-receptor bonds (Figure 2-B), each of which ruptures and binds stochastically.

Let 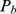 be the probability with which a single receptor binds to a ligand on the substrate during a time interval 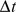.
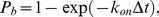
(7)

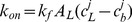
(8)where 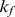 is the forward reaction rate (1 molecule^−1^ s^−1^), 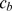 represents the density of bound ligands, 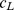 the original density of the ligands (molecules area^−1^), and 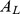 the area associated with the integrin node under consideration. Note that 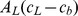 represents the number of unbound ligands available for bonding in the vicinity of the integrin node. In simulations, a triangular mesh of approximate side lengths of 0.75 µm were used for area 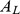. (See Figure S3).

Similarly, existing ligand-receptor bonds may rupture with probability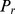 during a time interval 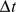,
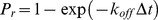
(9)where 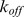 is the kinetic dissociation rate at a distance 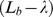 from the force equilibrium location. Here, 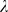 is the equilibrium distance of an integrin when it is unstressed (20–30 nm) [38], 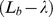 represents the stretched distance from the equilibrium (See Figure 2-B). We utilized the Bell's model to run stochastic simulation of bond rupturing and bonding, Bell's equation for the kinetic dissociation rate is defined by [39]

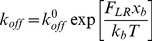
(10)where 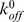 is the kinetic dissociation rate (1 s^−1^) under unstressed conditions with an equilibrium distance 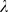, 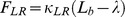 is a force applied to the bond, 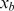 is the transition distance (0.02 nm), 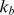 is the Boltzmann constant, and *T* is absolute temperature [39].

The number of ligand-receptor bonds, i.e. the size of each focal adhesion, can be simulated with these binding and rupture probabilities. Let 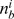 be the number of ligand-receptor bonds at the *i-th* integrin node, and 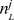 be the number of ligands on the *j-th* local surface near the *i-th* integrin node. The initial value of 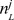 is calculated by multiplying 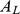 and 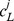. The number of bonds and available ligands vary stochastically. By drawing a random number, 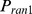, between 0 and 1:

If *P_ran_*_1_<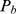, then one bonding occurs, update 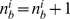 and 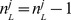.

Similarly, the rupture of ligand-receptor bonds can be simulated by drawing a random number, 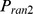:

If *P_ran_*_2_<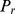, then one rupture occurs, update 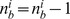 and 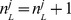.

Above bonding-rupture tests continue in subsequent time until the bond breaks completely (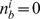).

Once 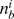 is known, the focal adhesion force of the *i*-th integrin node 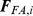 is computed as
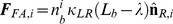
(11)where 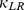 is an effective spring constant for a single ligand-receptor bond (∼1 pN/nm) [40], and 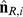 is a unit normal vector representing the *i*-th integrin node's direction on the cell membrane (See Figure 2-B). This focal adhesion force 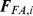 acts between the *i*-th integrin node and the point on the ECM surface where the extension of the unit normal vector 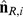 intersects with the ECM surface. From Figure 2-B this intersection position, that is, the root location of receptor and ligand bonds (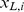), is given by
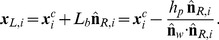
(12)where 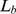 is the bond length, 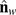 is the unit normal vector of the ECM surface, and 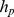 is the gap between the *i*-th integrin node and the ECM surface, as shown in Figure 2-B. These expressions are valid only when 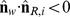 and the gap 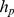 is less than a critical height (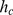) of 300 nm (<10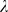 ): 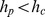. The latter condition is to restrict the formation of receptor-ligand bonds within the upper limit 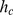.

### Comparison to 2-D cell migration experiments

The first set of cell migration simulations was aimed at comparing the integrated model against the experimental data published previously. Palecek et al. [17] performed CHO cell migration experiments in 2-D planar plates under various fibronectin coating concentrations. They found that the observed cell migration speed significantly depends on substratum ligand level, cell integrin expression level and integrin–ligand binding affinity. Interestingly, CHO cell migration speed exhibits a biphasic dependence on extracellular-matrix ligand concentration regardless of integrin expression level (the α_5_β_1_ receptor on fibronectin) [17]. The simulation results, too, showed similar behaviours of the biphasic dependence on fibronectin coating concentrations.

**Figure 3:**
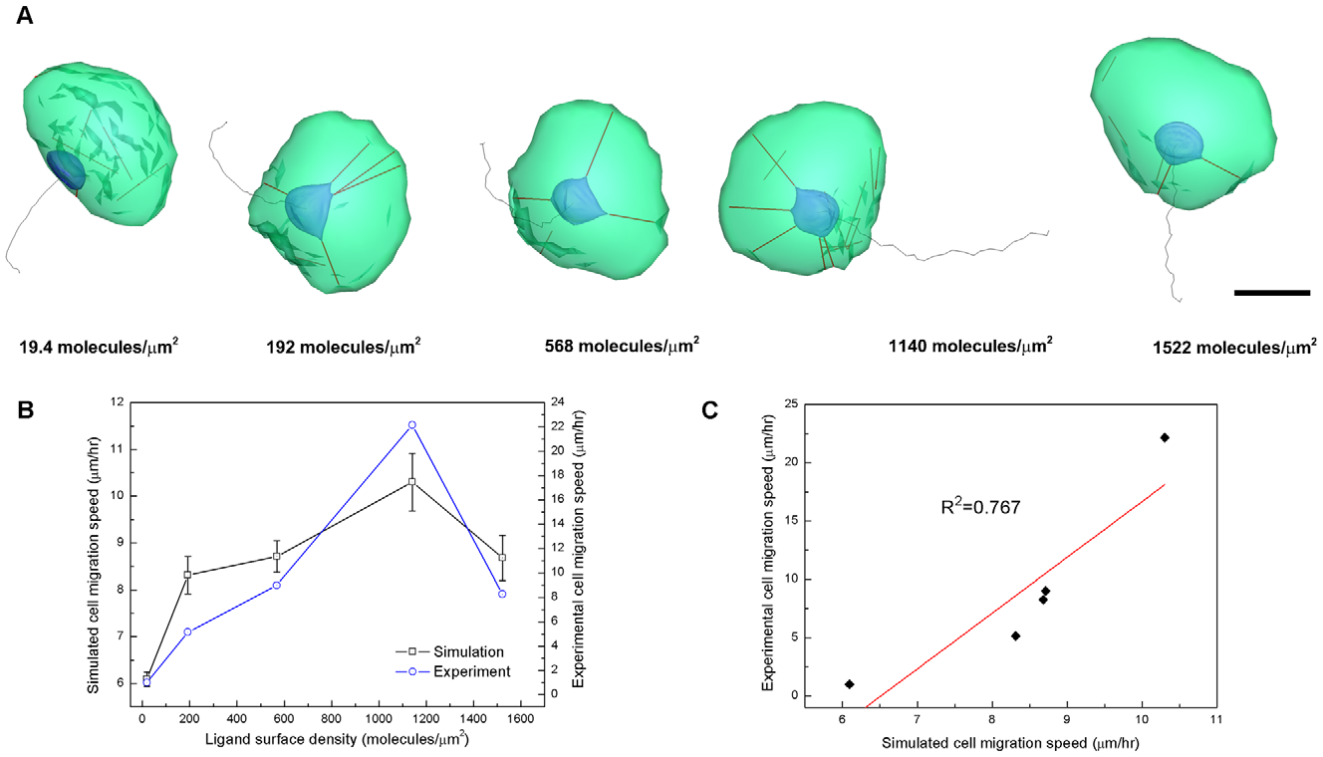
Cell migration along the planar surface of fibronectin. A) Simulated trajectories of cell migrations on fibronectin coated substrates under five different ligand surface densities of 19.4, 192, 568, 1140 and 1522 molecules/µm^2^. The black lines indicate trajectories of nuclei for the first three hours, B) comparison of average cell migration speeds: the simulation model vs. experiment data by Palecek *et al.* [17]. Average speed and standard error of mean (N = 5) are shown for the five different ligand surface densities, and C). linear regression (R^2^ = 0.767) of simulated migration speed vs. experimental migration speed.

Figure 3-A show samples of trajectories and morphologies of simulated cell migrations along the planar surface of five different fibronectin surface densities of 19.4, 192, 568, 1140 and 1522 molecules µm^−2^ for three hours (see Videos S1, S2, S3, S4 and S5). The ligand densities used for the simulations matched those of the available experiment data; ligand surface densities of fibronectin were converted from fibronectin plate concentrations (µg ml^−2^) using the relationship between plating concentration and ligand surface density of fibronectin [41]. First the total path length of each trajectory was obtained and was divided by the travelling time, 3 hours, to obtain the time-averaged cell migration speed. In the experiments, the speed of CHO cell migration was monitored in every 15 minutes, and was time averaged over the entire migration period (12 h) for each of fibronectin concentrations. Figure 3-B compares the average migration speed between the experiment and simulations. Here an error bar indicates a SE (standard error) of means.

The experimental data show that the cell migration speed is the lowest when migrating in the lowest ligand density, increases with increasing the ligand density, reaches a maximum value at the ligand density of 1140 molecules µm^−2^, and then decreases as the ligand density becomes too dense (Figure 3-B) [17], [41]. The simulated cell migration speed, too, shows a trend similar to the experiments: slow for a very low ligand density, the fastest at the particular ligand density of 1140 molecules µm^−2^, then slower again for the highest simulated ligand density. Both experiments and simulations attain the fastest speed at the particular ligand density of 1140 molecules µm^−2^. Overall both the simulation and experiment show an excellent agreement over the ligand density range of 10∼1500 [molecules µm^−2^]. Statistical analysis of linear regression was performed by comparing the experiment and the simulation in terms of the mean values of time-averaged cell migration speed for the same ligand density. As shown in Figure 3-C, good correlations were found between the two with *R* ^2^ = 0.767. Therefore, the model validates and, in turn, is validated by showing that cell migration speeds are strongly dependent on ligand density.

### Comparison to 2-D cell spreading experiments

**Figure 4:**
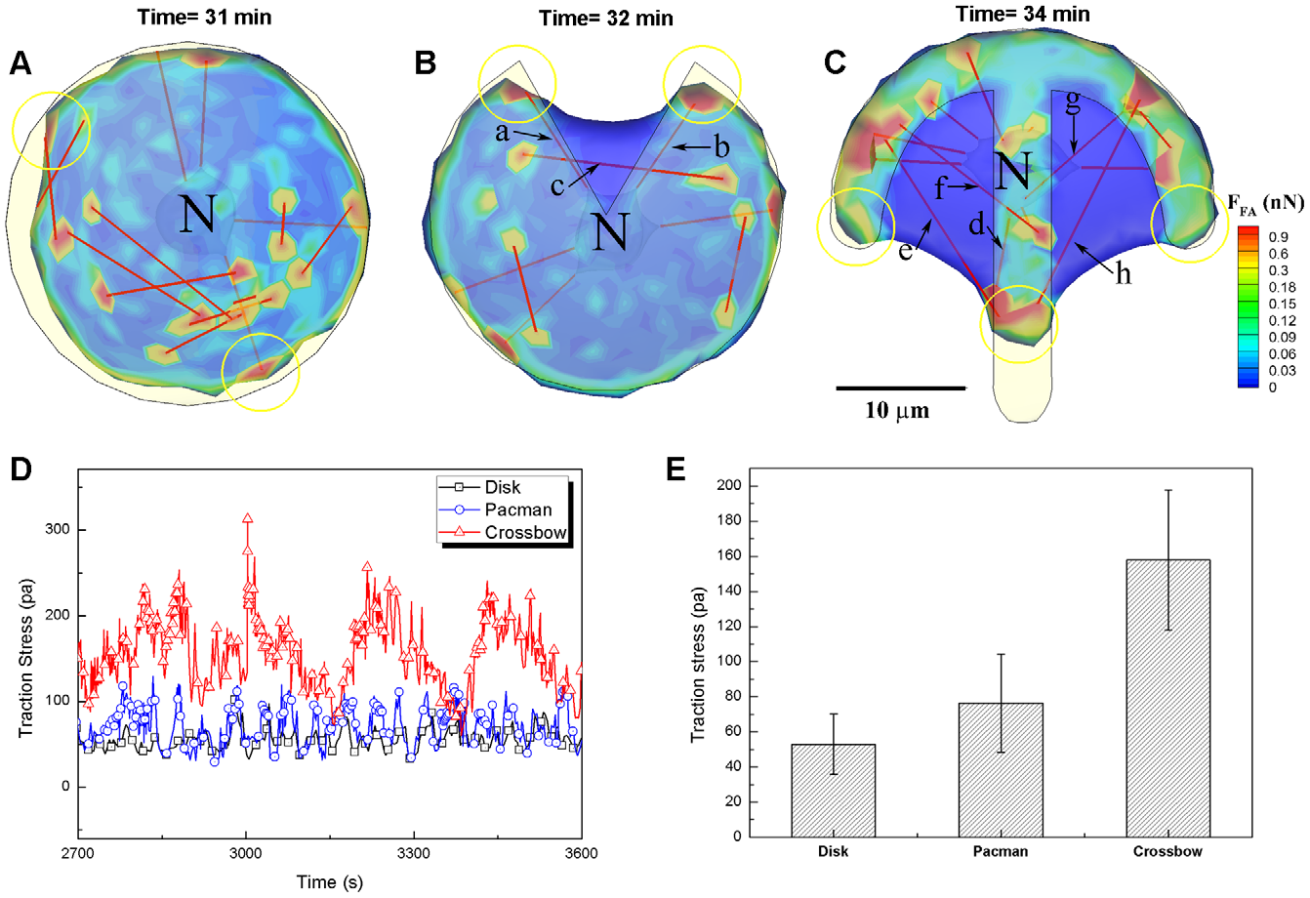
Contour plots of traction (or FA) force on ventral cell surfaces. Spreading cells on three fibronectin coated micropatterns of A) disk, B) pacman and C) crossbow shapes. Plots also reveal distributions of oriented ventral SFs and SFs connected to the nucleus (red lines). **N** indicates a nucleus and scale bar is 10 µm. D) Temporal variations of total traction stress per a cell on three different micropatterns, and E) time-averaged total traction stress of the cell for one hour is high in the order of the crossbow, pacman and disk shapes.

The second set of cell spreading simulation was intended to compare the integrated model against the recent experimental data published by Tseng *et al.* [18]. They developed a method to micropattern ECM proteins on poly-acrylamide gels in order to impinge on cell morphology and mechanics simultaneously, and have reported that measured traction forces differ considerably depending on the shape of micropatterns. In particular, in the case of the crossbow shaped micropatterns, concentrated cell traction forces are repeatedly located in the bottom part of the vertical bar. The simulation of the integrated model also showed similar spreading cell morphologies on micropatterned models and traction force distributions on the cell surface (Figure 4-A, B and C).

Figure 4 shows spreading cell morphologies with traction force contours and oriented SFs on three micropatterned geometries (a disk, a “pacman” shape, and a crossbow shape), after 60 minutes of spreading time for all shapes (see Videos S6, S7 and S8). Initially, all cell models start spreading from a spherical shape. The dimensions of micropatterns used for the simulations matches those of experiments for quantitative comparisons regarding contour plots of traction forces (or 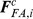) and spatial distributions of SFs inside of the cell; we obtained traction stress per a cell (unit: Pa) by dividing summations of tangential component of 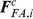 at *i*-th integrin node by a total area of ventral cell surface where focal adhesions are formed (Figure 4-D and E). Outside of the micropatterns, it was assumed that the ligand density was zero such that focal adhesion and lamellipodia protrusive forces only existed within the micropatterns.

Both experiments and simulations reveal similar trends in terms of concentrated traction forces on local areas of the ventral cell surface (Figure 4-A, B and C) as well as the order of higher traction stress per a cell among the three micropatterns (Figure 4-D and E). For the disk shaped micropattern, a few concentrated traction stress areas were observed at the ridge of the disk (Figure 4-A, two yellow circles). However, locations of concentrated traction forces on the disk shaped micropattern stochastically varied with time (see Video S6). This time-varying inconsistent distribution of stress on the pattern may be due to the smooth ridge of the shape, which gives a short length of receptor-ligand bonds such that the traction energy dissipates quickly. In the case of the “pacman” shaped micropattern, two sites of concentrated traction stress (Figure 4-B, two yellow circles) with SFs connected to the nucleus (Figure 4-B, black arrows a, b) and an oriented ventral SF was observed in between the sharp edges of the “pacman” mouth, as seen in experimental observations (Figure 4-B, black arrow **c**) although additional concentrated traction forces were located in the smooth ridge of the shape like the disk shaped micropattern. Interestingly, this behaviour was visualized to be persistent over time (see Video S7). In the case of the crossbow shaped micropattern, ventral SFs were aligned along the top roof and the bottom bar, as seen in experimental observations (Figure 4-C, black arrows e, f, g, h), and three sites of concentrated traction stress were observed at right and left end tips of the top roof and a bottom part of the vertical bar (Figure 4-C, three yellow circles). In addition, the strongest traction stress resulted from the contractile activity of SFs (Figure 4-C, black arrow d) at the bottom part of the vertical bar. As the activity of actin SFs are stronger, the length of receptor-ligand bonds is stretched more at the leading edge, which results in stronger traction stress. The animation of cell spreading simulation on the crossbow shaped micropattern, too, shows concentrated traction force at theses three sites (see Video S8).

Since a cell tends to migrate toward the stiffer gel region from the more compliant one [42], the cell may sense locally increased tension at the sharp edge of the micropatterns as the fibronectin bundles are anchored to the plate [43]. Thereby, larger areas of FAs are formed at the corners of the micropatterns while smaller areas of FAs are observed at the round boundary. From the agreement between simulation and experimental results on these micropatterned shapes, the model validates and, in turn, is validated by showing persistent high stress concentrations at sharp geometrically patterned edges.

## Discussion

### Coupling of focal adhesion dynamics and motor activity

**Figure 5:**
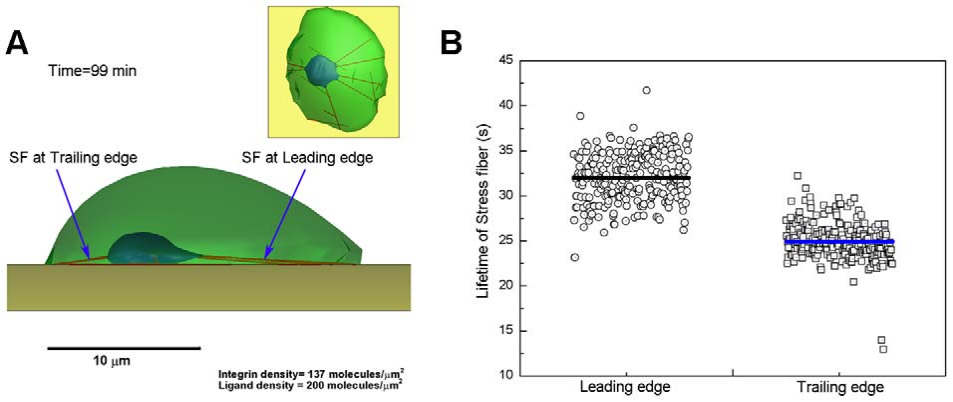
Actin motor activity in the model. A) An example of simulated cell migration on the plate showing that two types of stress fibers connected to the nucleus are anchored at both leading and trailing edges, and a schematic in the inset representing distributions of SFs in the cell in a top view. B) A scatter plot showing the lifetime of SFs at both leading and trailing edges. black and blue colored bold lines indicate averages values of 32.00 s and 24.91 s at the leading and trailing edges, respectively. Statistical data were acquired from 266 focal adhesions sites at the leading edge and 245 focal adhesions sites at the trailing edge during 3 hours of simulated cell migration on the plate.

It has been reported that nascent adhesions (smaller than ∼0.25 µm) initiate the adhesion of protrusions of the leading edge of the cell, followed by the disassembly of a subpopulation of nascent adhesions within a minute and growth of the remainder into focal complexes (∼0.5 µm in size) and then focal adhesions (1–5 µm in size) within 5 minutes [44]. Afterwards, focal adhesions either disassemble or mature within the ventral surface of the cell membrane within 10–20 minutes [45], [46]. Furthermore, it is known that the maturation and turnover of focal adhesions involves protein recruitment and elongation, followed by protein disengagement and shrinkage [46]. In the current integrative cell migration model, the disengagement of actin stress fibers from integrins bound to the ECM is assumed to occur when a force-transmitting structural linkage ruptures (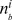 = 0) (see Figure 2-B). With the onset of motor activity after actin polymerization, the generated force is transmitted to the focal adhesions, and receptor-ligand bonds at the focal adhesions are subsequently stretched, resulting in an increases in both traction force and rupture probability for a receptor-ligand bond according to Bell's law [39]. As shown in Figure 5-A, the situation differs at the leading and trailing edges, in large part due to the location of the nucleus closer to the rear of the cell. Note that the angle between the inclined stress fiber and the horizontal plane of the substrate at the trailing edge is higher than that at the leading edge of the cell. If we assume that the stress fibers all exert comparable levels of force then the normal force component will be larger at the trailing edge and therefore have a higher probability of rupture, thereby allowing forward motion of the cell. To test this hypothesis, 266 stress fibers connected to the nucleus at the leading edge and 245 stress fibers connected to the nucleus at the trailing edge were monitored and statistically analysed during three hours of simulated cell migration on the plate with fibronectin density of 200 molecules/µm^2^ (Figure 5-A, Video S9). Consistent with this hypothesis, we found the lifetime of stress fibers at the trailing edge to be less than that at the leading edge of the cell; 32.00±2.78 s at the leading edge and 24.92±2.17 s at the trailing edge (Figure 5-B). Therefore, we propose that increased magnitude of normal force on the adhesion site at the trailing edge plays a key role in accelerating the rupture of receptor-ligand bonds, leading to an increase in cell migration speed.

### Lifetime of actin stress fiber

Our modelled stress fiber lifetime physically represents a contractile SF period which is related with the turnover time of the three main dynamic components consisting of SF-actin, alpha-actinin, and myosin. However, it should be noted that there is total lifetime of stress fiber which includes multiple periods of the lifetime of its constituent until it fully disappears. Recently, Hotulainen and Lappalainen [47] have observed highly dynamic associations and dissociations of these components in the SF by FRAP analysis. They found recovery times for actin, alpha-actinin, and myosin light chain (MLC) in bleached regions of the SF were 323 s, 123 s, and 223 s, respectively (see fig 7A in [47]). Interestingly, all components of the SF (see fig. 7A in [47], white boxes) disappeared at the time of +4 s (depolymerization occurs) after SF's contractile motion got started at the time of −20 s. Thus, it seems to us that this time period of 24 s may be related with contractile period of the SF among full periods of the SF (actin polymerization, SF contractile motion, and actin depolymerization). Additionally, time periods for actin polymerization and actin depolymerization in our model were set to be 180 s and 1–5 s, respectively, and time period for SF contractile motion in the model was determined to be ∼30 s. Summation over the full period yields ∼215 s, which is within a similar range of the recovery times for the three main components of a SF.

It should be noted that most nonmotile cell types contain thick, non-dynamic stress fibers, whereas most motile cell types contain very few and thin stress fibers [47] or few and large stress fibers on the soft substrata [48]. In case of nonmotile cells, most SFs are known to form at the ventral surface of the cell, and its movements are very slow. However, in case of motile cells, it is possible to assemble ventral SFs by the interaction with preassembled dorsal SFs and transverse arcs within the period of 27 min (see fig.5 in [47]). During the course of the assembly of ventral SF in motile cells, three major processes (actin polymerization, SF contractile motion, and actin depolymerization) are periodically repeated due to the turnover of actin in either dorsal SF or transverse arcs and SFs' alignments were dynamically varied due to actin motor activity. Thus, it should be emphasized that there exist three main highly dynamic processes of the SF. In addition, it has been known that rapid SF depolymerization occur because of cell shortening [49] or SF detachment via localized application of trypsin at focal adhesions [50], [51].

Note that for the sake of video visualization of the processes of actin polymerization and bundling, the frame-to-frame time scale is 360 s while the simulation time step used is 0.001–0.01 s. Because the frame rate is greater than the SFs dynamic period (∼215 s), the simulated SF dynamics may appear discontinuous, when they are, in fact, not.

### What is important to maximum cell migration speed?

Although there are differences in cell migration speeds between the model and experiment, we are interested in similar trends across a range of the ligand density, and linear regression between the cell migration speed of both the model and experiment with identical ligand density confirms good agreement between the model and experimental data. Additionally, we also simulated cell migration models in which SFs are disconnected from the nuclear membrane on the substrates under five ligand surface densities (Figure S4), which resulted in lower cell migration speed than cell migration model with SFs connected to the nuclear membrane (Figure 3-B). Thus, our simulated results reveal that these SFs connected to the nucleus play an important role in cell migration. In the literature [30], the authors also demonstrated that nesprin-1 depleted endothelial cells showed decreased migration speed with no SFs connected to the nuclear membrane. Furthermore, Khatau, *et al.* [43] highlighted the interplay between cell shape, nuclear shape, and cell adhesion mediated by the perinuclear actin cap. We also found that the cell migration speed is limited by ligand density and integrin density (Figure S5). They work together to promote adhesion of the cell, and in turn, cell speed. This example shows how either value alone is enough to act as a bottle neck and limit the migration speed. If the ligand density is high (950 molecules/µm^2^), but the integrin density is insufficient (≤137 molecules/µm^2^), the cell speed will be limited. Similarly, if the integrin density is high (205 molecules/µm^2^) but the ligand density is insufficient (200 molecules/µm^2^), then the migration speed is again limited (Figure S5). We believe that the integration of focal adhesion dynamics (receptor-ligand bonds) and actin motor activity is important to observe and predict maximum cell migration speeds. In addition, as cell's contacting area on the substrate becomes larger, the numbers of focal adhesion sites such that ventral SFs anchored at FAs is increased. That is to say, two resultant forces from focal adhesions and actin SFs are increased and they are important to capture the maximum cell migration speed dependent on substrate geometry as well as ligand surface density.

**Figure 6:**
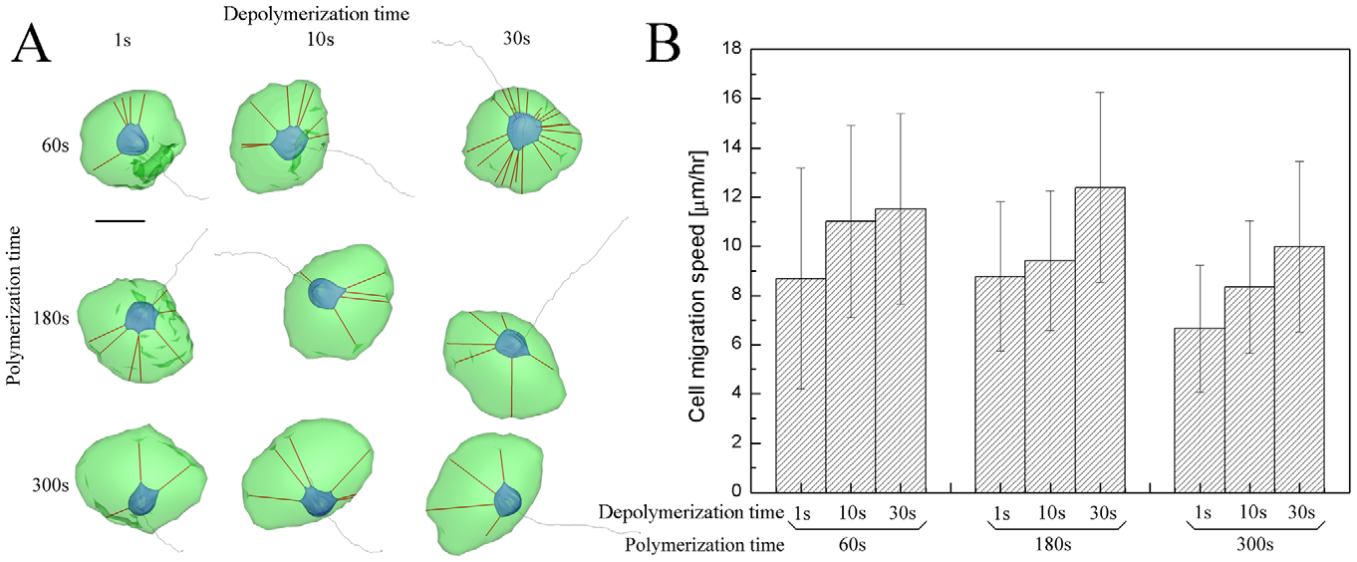
Optimal condition of cell migration. A) Trajectories and morphologies of simulated cell migrations along the planar surface of fibronectin surface density of 1140 molecules/µm2 for three hours under nine different cases of polymerization times with 60, 180, and 300 s (rows) and depolymerization times with 1, 10, and 30 s (columns), and B) bar graphs showing time-averaged cell migration speeds and error bars indicate standard deviations for nine different cases in A). Scale bar is 10 µm.

Figure 6-A shows samples of trajectories and morphologies of simulated cell migrations along the planar surface of fibronectin surface density of 1140 molecules/µm^2^ for three hours under nine different cases of polymerization times with 60, 180, and 300 s (rows) and depolymerization times with 1, 10, and 30 s (columns). First, simulated data were compared with different depolymerization times for the three values (rows) of polymerization times of 60, 180, and 300 s. Cell migration speed at each value (row) of polymerization time increases as the depolymerization time becomes larger (Figure 6-B). In the case of the polymerization time of 60 s, especially, the morphologies of cells were observed to be round. This phenomenon results from faster actin motor activity with the inclusion of a shorter polymerization process. Thereby, the occurrence of more frequent actin motor activity prevents the cell from stretching more than the other cases of polymerization times of 180 and 300 s. On the other hand, as the polymerization time becomes larger, the cell tends to stretch more and its morphology is changed to wider crescent-shape from the rounded shape. Next, simulated data were compared with different polymerization times for three values (columns) of depolymerization times of 1, 10, and 30 s (Figure 6-B). As for cases of depolymerization times of 1 and 10 s, cell migration speed increases as polymerization time decreases. In our model, a shorter polymerization process represents faster FA component (integrin and vinculin) renewal within FAs due to increased level of myosin II activation per FA. Contraction could pull these components out of FAs. It has been reported that faster turnover rates of vinculin and integrin due to further increase in actomyosin contractility are correlated with faster cell migration speed at the intermediated ligand surface density [45]. However, in case of depolymerization time of 30 s, cell migration speed takes a maximum at an intermediated value of polymerization time of 180 s, which suggest that a balance between adhesion strength and myosin II activity is required for optimal cell migration [45].

## Model

### Membrane stiffness and elastic forces

The elastic forces, 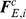 and 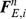, are obtained by using the virtual work theory in structural mechanics. To this end, the elastic energy stored in the cell and nucleus membranes are obtained. Two types of elastic energy are considered. One is the elastic energy associated with distance changes between surface nodes [52], [53]:
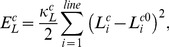
(13a)

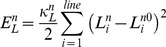
(13b)where 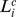 is the length of the *i*-th line of the cell membrane mesh, and 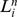 is that of the nucleus. Both are updated at every time-step. 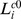 and 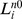 are their relaxed (zero force) lengths. 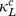 and 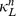 are effective stiffness constants of the line elements of the cell membrane (5.0×10^−5^ N/m) [32], [54] and nucleus (5.0×10^−3^ N/m) [55], respectively. Similarly, the elastic energy associated with area changes is given by
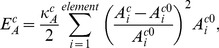
(14a)

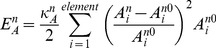
(14b)where 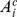 is the *i*-th mesh area of the cell membrane and 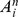 is that of the nucleus. 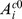 and 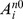 are their relaxed values. Parameters 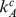 and 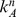 are effective stiffness constants of area elements of the cell membrane (1.0×10^−4^ N/m^2^) and nucleus (1.0×10^−4^ N/m^2^), respectively [53].

Elastic forces 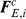 and 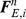 can be obtained by differentiating the total energy,
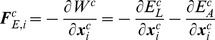
(15a)

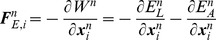
(15b)where 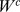 and 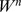 indicate total stored energies of the cell membrane and nucleus, respectively, and 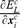, 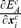, 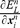 and 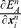 are obtained analytically.

### Actin motor activity

An actin SF is a bundle of actin microfilaments assembled by actin-myosin II interactions. It is known that at least one end of each SF is connected to focal adhesion molecules, such as vinculin, talin, paxillin, zyxin, and FAK [38], and the other end of a SF can be connected to the nuclear membrane [30], transmitting a force to the nucleus. In the model, the *i*-th integrin node is connected to the *j*-th nuclear node by a SF. Its connection to the *j*-th nuclear node is determined by the nearest distance from the *i*-th integrin node to the nucleus. In addition, the *i*-th integrin node is connected to the *k*-th integrin node by a ventral SF. To consider the alignment of the ventral SF which is preferentially parallel to the stronger elastic resistance direction [42], [56], its connection to the *j*-th integrin node is established by the lower principal direction of Lagrange strain tensor [57] at the cortical surface bound to the *i*-th integrin node.

The stiffness of a SF is variable. According to the literature, the stiffness increases with a contractile agonist (histamine) and decreases with a relaxing agonist (isoproterenol) [58]. These characteristics must be reflected in the formulation of the SF stiffness:
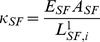
(16)where 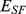 is Young's modulus of SFs (230 kPa) directly measured from isolated smooth muscle cells [59], 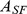 is the average cross-sectional area of SFs (250 nm in radius [60]), and 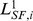 is the length of a single compartment of the *i*-th SF. As shown in Figure 2-B, a SF consists of 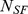 contractile compartments, each of which consists of two half ‘I bands’ (F-actin filaments) and an ‘A band’ (myosin II) in F-actin filaments [61], [62]. 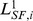 represents the unstressed length of the *i*-th contractile compartment, which slides at a rate 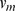 at both ends. Therefore,
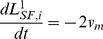
(17a)

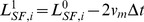
(17b)where equation (17b) is the discretized form of equation (17a), and 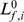 indicates the length of a single unit of the *i*-th SF at the previous time (*t*−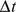) [63]. Similarly, the elastic energy stored in the *i*-th SF is given by
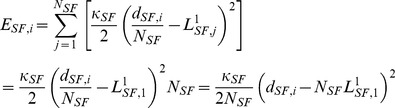
(18a)where 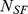 is the number of contractile compartments in the *i*-th SF, 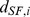 represents the distance between *i*-th integrin and *j*-th nuclear nodes for a SF connected to the nucleus or between *i*-th integrin and *j*-th integrin nodes for a ventral SF. It should be noted that 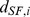 physically means the length of SFs under tension and 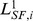 represents the length of a single unstressed bundle of SFs (See Figure 2-B). Using the virtual work theory, forces due to actin SFs' motor activity at the *i*-th integrin and *j*-th nuclear nodes or at *i*-th integrin and *j*-th integrin nodes (ventral SFs) are given by
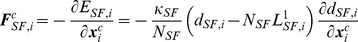
(18b)

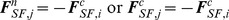
(18c)These forces are generated when focal adhesions have been formed and F-actin filaments are fully polymerized. It has been known that SF assembly occurs over several minutes [64]–[66], but SF disassembles rapidly within seconds [67]–[68]. In addition, it takes several minutes to form FAs from focal complexes (FCs). These observations suggest that myosin motor activities in SFs are switched off during the remodelling of the actin cytoskeleton (polymerization) and SF turnover. In our simulations, time for full formation of F-actin is set to be 180 s, and time for the complete disassembly of F-actin is set to 1 s, based on the above reference information.

Actin motor activity is assumed not to start until the other end of a SF is connected to the nucleus. Time for polymerization of F-actin appears to be the waiting time before actin motor activity takes place, during which time an adhesion complex (AC) becomes a fully developed FA. The myosin II's sliding rate is known to fluctuate (i.e. is non-uniform) unlike myosin I which slides with a uniform rate. Furthermore, the sliding rate of myosin II is adjusted by sensing the transmitted focal adhesion force from the ECM [23]. To incorporate these characteristics into the model, force-velocity relation of muscle myosin II, first proposed by A.V. Hill [69], is adopted as the following equation:
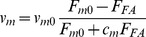
(19)where 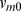 is the sliding rate of myosin in the absence of load (10 nm/s) [63], 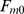 is the isometric force of myosin, or stall force, and 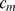 is a parameter for the force-velocity relationship for myosin. Initially, the length of sarcomere unit is 800 nm (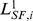 = 800 nm at *t* = 0 s), which contracts until 60% of the initial length has contracted. As the contraction takes place at both sides of each sarcomere unit, the minimum time required for 60% contraction is calculated as 16 s with 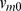. Furthermore, an additional condition for terminating actin motor activity is also considered when integrin nodes are broken from FA formations. Afterwards, the depolymerization of actin SFs occurs in 1 s. During this period, formations of nascent ACs are inhibited. In summary, actin motor activity consists of three evolving periods, polymerization (180 s), motor activity (>16 s) and depolymerization (1 s) [64]–[68].

### Lamellipodium force

Lamellipodium force is a characteristic feature at the leading edge of migratory cells. It is believed to be the motor which push the cortical cytoskeleton forward during the process of cell migration. Normally, cells experience a small protrusive pressure that results from osmotic pressure or actin branches stimulated by activated arp2/3 [70]. Recently, time-averaged high protrusive force measured per pillar was 800 pN for NIH 3T3 fibroblasts and diseased cells [71]. Here, we assume lamellipodium protrusive force is due to constant actin polymerization rate [72]. Thereby, we approximate the magnitude of the lamellipodium force at the *i*-th integrin node (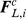) is constant at 300 pN and exists at only leading edges of the cell. It should be noted that the magnitude of net force at the *i*-th integrin node is non-uniform because it is a vectorial sum of 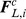 and the local membrane restoring forces from neighboring nodes.

### Numerical methods of “integrated cell migration model”

Cell migration simulations were carried out using a fourth order Rosenbrock method [73] based on an adaptive time-stepping technique for integrating ordinary differential equations with the convergence criterion <10^−4^. The ordinary differential equations were solved for the 6×*N* (*N* = 549 for both cell migration and spreading simulations) unknown variables associated with the mesh node position vectors for both cell membrane and nucleus membrane: 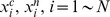 (see Figure 2-A). For cell migration simulation the Rosenbrock method outperforms the standard Runge–Kutta method which requires a relatively large number of iterations [73]. Furthermore, the Rosenbrock method consumes less computing time by using adaptive time-step control that ranges from 10^−3^ s to 10^−2^ s in the present work. Thus, it is suitable for simulating transient cell migratory behaviours over 10 hours.

The focal adhesion dynamics were computed based on the Monte-Carlo simulation. The model assumes a total of 164,700 integrin molecules on the cell membrane [74] and 549 integrin nodes for both cell migration and spreading models with a cell radius of 8 µm. Therefore, the density of receptors over the cell membrane is 300 integrins/node for both models, among which some fraction of integrins bond to ligands; the number of ligand-receptor bonds varies stochastically in the range 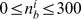. Recall that 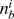 is determined by drawing random numbers 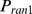 and 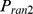 and simulating binding and rupturing events stochastically using Bell's equation. Additionally, each integrin node represents a collection of integrins having the collective stiffness 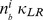 for 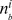 receptor-ligand bonds (see equation (11)).

The elastic force at the *i*-th node 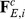 represents the resultant force acting on the *i*-th node that is calculated by vectorial addition of elastic forces from neighbouring nodes. To compute this, first the coordinates of each node are updated in each time cycle, and distances from each node to neighbouring nodes are computed along with the areas of the surrounding rectangles. The elastic forces are derived from these distances and areas for individual nodes.

The methods for building geometrical models for the simulation of cell migration have been well documented in the literatures [75], [76]. See also geometrical models of micropatterns, as shown in Figure S3. One practical issue in computing finite mesh geometric models is to check geometrical compatibility. As the coordinates of cell membrane and nuclear nodes are updated based on the equations of motion, geometrically incompatible situations occur occasionally in the configurations of the cell membrane mesh and that of the nucleus in relation to the curved ECM surface. For example, some cell membrane nodes intersect with the substrate, and the nucleus intersects with the cell membrane. These incompatible situations must be checked in every computational cycle, and necessary corrections must be made.

## Supporting Information

Figure S1 Schematics of A) 2-D cell migration in planar surface, B) 2-D cell migration and spreading on a micropatterned structure, C) 3-D cell migration in a rectangular channel and D) 3-D cell migration in 3-D ECM.
(TIF)

Figure S2 Samples of A) cell migration speed and B) cell migration acceleration for three hours. Blue lines indicate time-averaged cell migration speed and acceleration of 4.24 nm/s and 3.18×10^−4^ nm/s^2^, respectively.
(TIF)

Figure S3 Meshes of three micropattern models of A) disk, B) pacman and C) crossbow shapes; all meshes have triangular elements with approximate side lengths of 0.75 µm.
(TIF)

Figure S4 Comparison of average cell migration speeds: cell migration model with SFs connected to the nuclear membrane vs. cell migration model with SFs disconnected to the nuclear membrane. Average speed and standard error of mean (N = 5) are shown for the five different ligand surface densities.
(TIF)

Figure S5 Comparisons of average cell migration speeds: cell migration model with four different integrin densities of 34, 68, 137, and 205 molecules/µm^2^ on the cell surface on two different low and high ligand surface densities of 200 and 950 molecules/µm^2^. Average speed and standard error of mean (N = 5) are shown for the four different integrin surface densities and two ligand surface densities.
(TIF)

Text S1 Why the net force is zero in a dynamic moving system?
(DOCX)

Video S1 Example of a simulated cell migration on the plate with the ligand density of 19.4 molecules/µm^2^. Cell and nuclear membranes are visualised with green and blue, respectively. Bold red lines in the cell indicate actin stress fibers, and black line indicates a trajectory of nuclear center. Six seconds of the video represents three hours.
(AVI)

Video S2 Example of a simulated cell migration on the plate with the ligand density of 192 molecules/µm^2^. Cell and nuclear membranes are visualised with green and blue, respectively. Bold red lines in the cell indicate actin stress fibers, and a black line indicates a trajectory of the nucleus center. Six seconds of the video represents three hours.
(AVI)

Video S3 Example of a simulated cell migration on the plate with the ligand density of 568 molecules/µm^2^. Cell and nuclear membranes are visualised with green and blue, respectively. Bold red lines in the cell indicate actin stress fibers, and a black line indicates a trajectory of the nucleus center. Six seconds of the video represents three hours.
(AVI)

Video S4 Example of a simulated cell migration on the plate with the ligand density of 1040 molecules/µm^2^. Cell and nuclear membranes are visualised with green and blue, respectively. Bold red lines in the cell indicate actin stress fibers, and a black line indicates a trajectory of nucleus center. Six seconds of the video represents three hours.
(AVI)

Video S5 Example of a simulated cell migration on the plate with the ligand density of 1522 molecules/µm^2^. Cell and nuclear membranes are visualised with green and blue, respectively. Bold red lines in the cell indicate actin stress fibers, and a black line indicates a trajectory of nucleus center. Six seconds of the video represents three hours.
(AVI)

Video S6 Example of a simulated cell spreading on the disk shaped micropattern with the ligand density of 475 molecules/µm^2^. Cell and nuclear membranes are visualised with green and blue, respectively. Bold red lines in the cell indicate actin stress fibers, and contours indicate traction forces on the ventral surface of cell membrane. Twelve seconds of the video represents sixty minutes.
(AVI)

Video S7 Example of a simulated cell spreading on the pacman shaped micropattern with the ligand density of 475 molecules/µm^2^. Cell and nuclear membranes are visualised with green and blue, respectively. Bold red lines in the cell indicate actin stress fibers, and contours indicate traction forces on the ventral surface of cell membrane. Twelve seconds of the video represents sixty minutes.
(AVI)

Video S8 Example of a simulated cell spreading on the crossbow shaped micropattern with the ligand density of 475 molecules/µm2. Cell and nuclear membranes are visualised with green and blue, respectively. Bold red in the cell lines indicate actin stress fibers, and contours indicate traction forces on the ventral surface of cell membrane. Twelve seconds of the video represents sixty minutes.
(AVI)

Video S9 Example of a simulated cell migration on the plate with the ligand density of 200 molecules/µm^2^. Cell and nuclear membranes are visualised with green and blue, respectively. Bold red lines in the cell indicate actin stress fibers. Twenty seconds of the video represents three hours.
(WMV)
