# Supplementary figures and images for: Dynamic Modeling of Cell Migration and Spreading Behaviors on Fibronectin Coated Planar Substrates and Micropatterned Geometries
Source: PLoS Comput Biol. 2013 Feb 28;9(2):e1002926. doi: 10.1371/journal.pcbi.1002926 (PMC3585413; doi:10.1371/journal.pcbi.1002926)

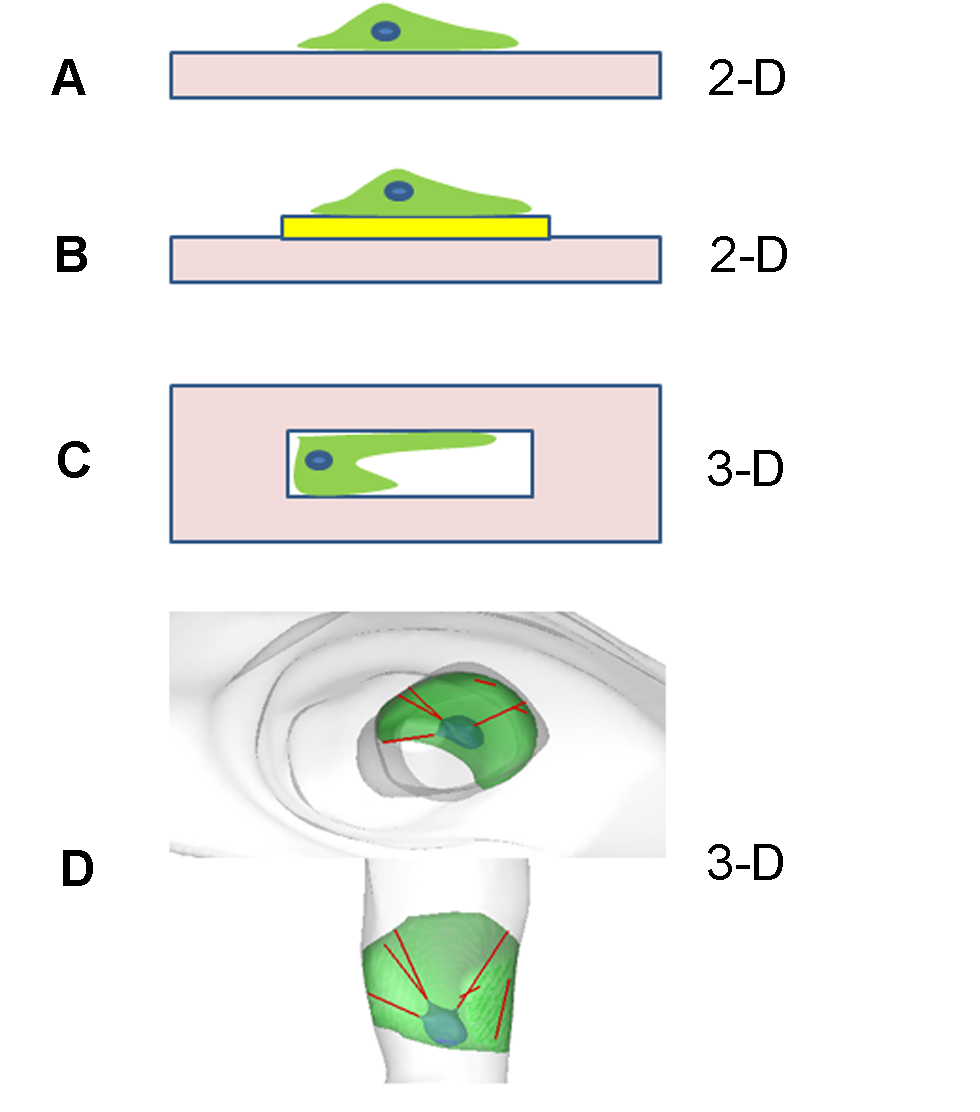

Supplement: Figure S1 — Schematics of A) 2-D cell migration in planar surface, B) 2-D cell migration and spreading on a micropatterned structure, C) 3-D cell migration in a rectangular channel and D) 3-D cell migration in 3-D ECM. (TIF) [file pcbi.1002926.s001.tif]

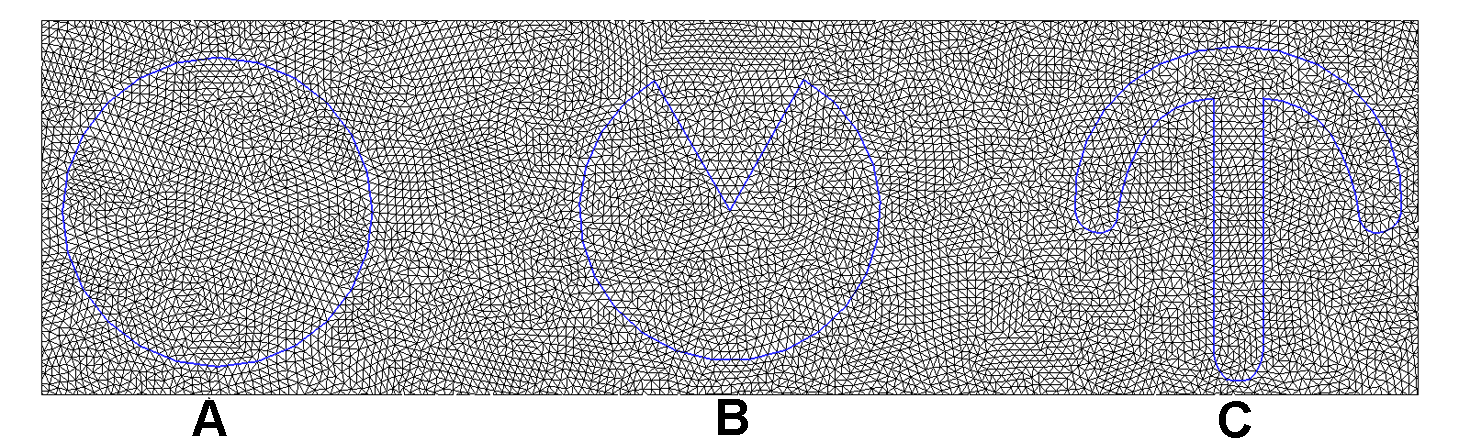

Supplement: Figure S3 — Meshes of three micropattern models of A) disk, B) pacman and C) crossbow shapes; all meshes have triangular elements with approximate side lengths of 0.75 µm. (TIF) [file pcbi.1002926.s003.tif]

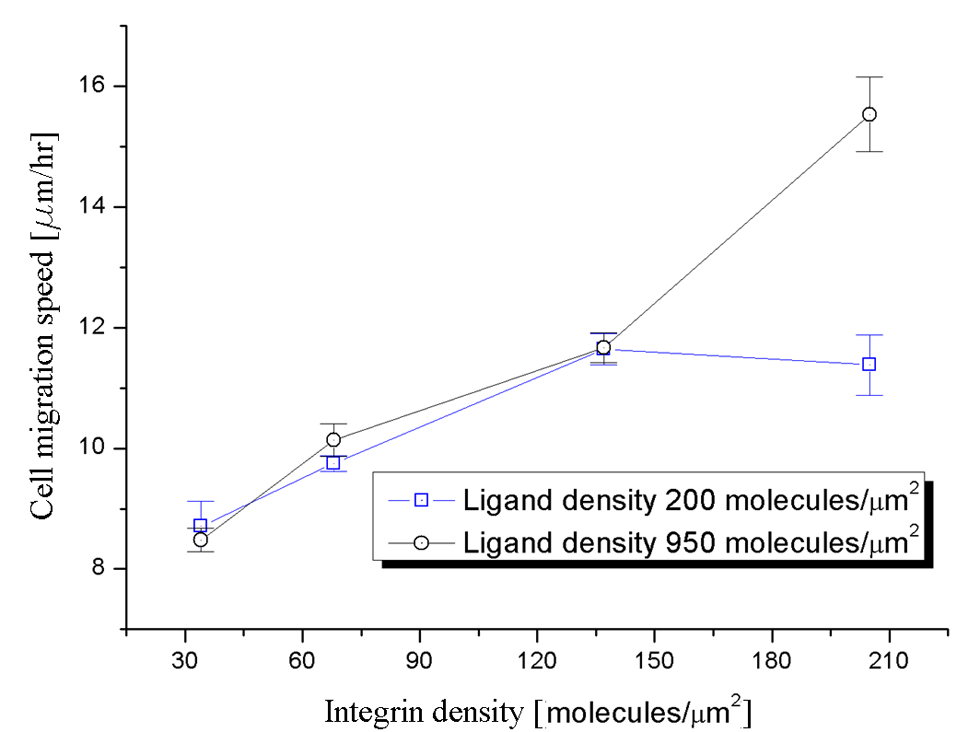

Supplement: Figure S5 — Comparisons of average cell migration speeds: cell migration model with four different integrin densities of 34, 68, 137, and 205 molecules/µm2 on the cell surface on two different low and high ligand surface densities of 200 and 950 molecules/µm2. Average speed and standard error of mean (N = 5) are shown for the four different integrin surface densities and two ligand surface densities. (TIF) [file pcbi.1002926.s005.tif]
