# Supplementary material for: Dynamic Modeling of Cell Migration and Spreading Behaviors on Fibronectin Coated Planar Substrates and Micropatterned Geometries
Source: PLoS Comput Biol. 2013 Feb 28;9(2):e1002926. doi: 10.1371/journal.pcbi.1002926 (PMC3585413; doi:10.1371/journal.pcbi.1002926)
Supplement: Text S1 — Why the net force is zero in a dynamic moving system? (DOCX) [file pcbi.1002926.s006.docx]

**Text S1**

**Why the net force is zero in a dynamic moving system?**

We calculated $m_{i}^{c}$, $v_{i}^{c}$,$\frac{dv_{i}^{c}}{dt}$ and $F_{D,i}^{c}$, and compared approximated value of the inertia term ,$m_{i}^{c}\frac{dv_{i}^{c}}{dt}$, with selected external forces terms of $F_{D,i}^{c}$ and $F_{L,i}^{c}$ in the following:

$m_{i}^{c}=\rho_{cell}V_{i}^{c}=\rho_{cell}\frac{4\pi}{3 N}R_{cell}^{3}\approx4.062\times10^{-15}kg$ (S1)

where $\rho_{cell}$is a density of the cell (1040 [kg/m^3^]), $V_{i}^{c}$ is a volume of spherical section, which is divided by total number of nodes (*N*=549), and *R* is a radios of spherical cell (8 μm).

Time-averaged $v_{i}^{c}$ and $\frac{dv_{i}^{c}}{dt}$ for three hours are calculated as 4.24 nm/s and 3.18×10^-4^ nm/s^2^ (**Figure S2**) respectively. $m_{i}^{c}\frac{dv_{i}^{c}}{dt}\approx1.292\times10^{-27}[N]$, $F_{L,i}^{c}$=300×10^-12^ [N], $F_{D,i}^{c}$=$-C_{c} v_{i}^{c}\approx4.24\times10^{-12}[N]$.

Thus, we found $m_{i}^{c}\frac{dv_{i}^{c}}{dt}$<<$F_{L,i}^{c}$ or $m_{i}^{c}\frac{dv_{i}^{c}}{dt}$<<$F_{D,i}^{c}$. In this approach, we assumed $m_{i}^{c}\frac{dv_{i}^{c}}{dt}\approx0$. Thereby, the net force is zero in a dynamic moving system.
